# *Notes from the Field:* Exposures to Chemical Munitions During Commercial Fishing Operations — New Jersey, 2016–2023

**DOI:** 10.15585/mmwr.mm7508a3

**Published:** 2026-03-05

**Authors:** Ryan Snead, Marija Borjan, Virginia Wheatley, Katharine McGreevy, Danielle Mills

**Affiliations:** ^1^New Jersey Department of Health; ^2^Epidemic Intelligence Service, CDC; ^3^Division of Environmental Health Science and Practice, National Center for Environmental Health, CDC.

**Summary**

**What is already known about this topic?**

Until 1970, unexploded chemical warfare munitions (CWMs), including sulfur mustard (mustard gas) from World War I and World War II, were disposed of at sea. Commercial fishing vessels occasionally inadvertently dredge sea-disposed CWMs, exposing workers and risking health and safety.

**What is added by this report?**

Three incidents of recovered CWMs in New Jersey waters occurred in 2016, 2017, and 2023, resulting in severe worker injuries and large-scale food product destruction.

**What are the implications for public health practice?**

The risk for inadvertent recovery of sea-disposed CWMs continues while munitions remain on the seafloor. Prioritizing avoidance of documented dump sites followed by engineering and administrative measures, interagency coordination, training, and use of personal protective equipment are recommended to mitigate the risk for future injuries and food contamination.

Until 1970, an estimated 17,000 tons of unexploded World War I and World War II chemical warfare munitions (CWMs), weapons designed to disperse toxic chemical agents to cause mass casualties or death, were disposed of off the U.S. Atlantic coast (*1*). Dredging, a commercial fishing method used to harvest seafloor species, occasionally results in the unintentional recovery of CWMs. These events have resulted in severe worker injuries (Figure) and potential food contamination (*2*,*3*). Three events associated with recovered CWMs that caused injuries or food contamination occurred in the mid-Atlantic and New England during 2004–2012 (*2*); this report describes three events that occurred off the New Jersey coast in August 2016, August 2017, and October 2023.

**FIGURE:**
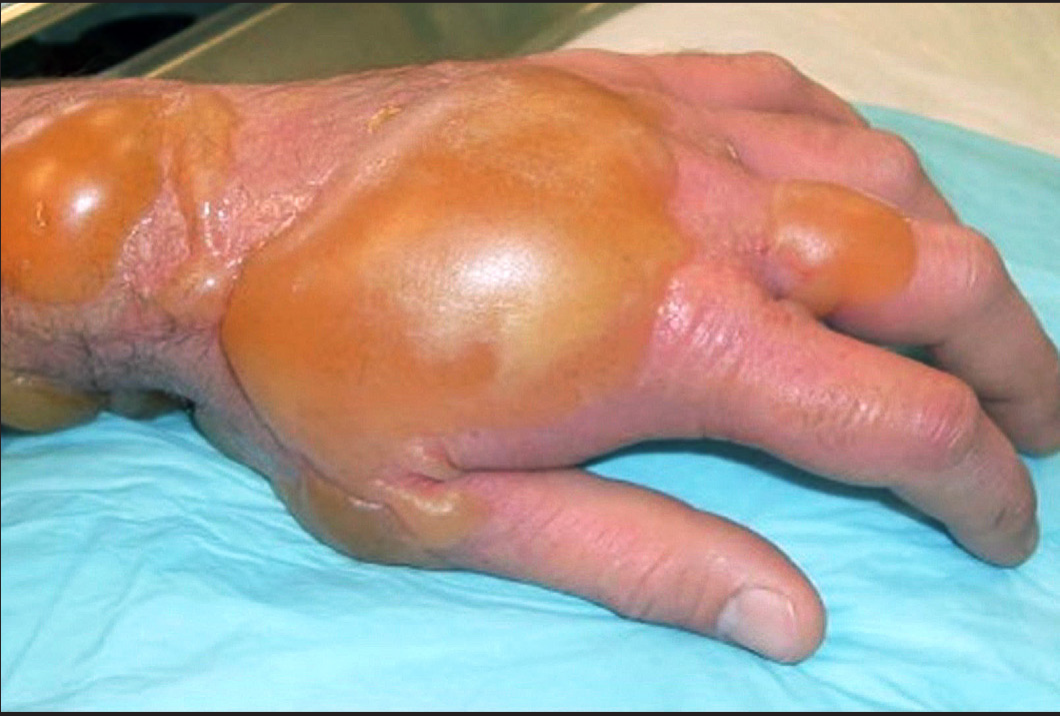
Example of injury to skin exposed to sulfur mustard–containing chemical warfare munitions* Photo/U.S. Air Force * This photograph is an example and does not depict any persons described in this report.

## Investigation and Outcomes

### Data Source and Analysis

Event details, including CWM specifics and handling, means of exposure, nature of injury and treatment, contamination and destruction of food, and vessel environmental response, were summarized by reviewing medical and billing records, poison control intake notes, correspondence with state and federal agencies, and information obtained from crewmembers. This activity was reviewed by CDC, deemed not research, and was conducted consistent with applicable federal law and CDC policy.*

Six crewmembers were exposed in the three events; all documented injuries were consistent with exposure to mustard agent (sulfur mustard), a chemical warfare vesicant that causes blistering of the skin and mucous membranes on contact. For each incident, information about all affected crewmembers is described. In all cases, shellfish processing plants fully cooperated during food destruction.

## Exposure Events

**August 2016.** A commercial fishing vessel dredged a ruptured CWM off the coast of Atlantic City. When discovered on the conveyor belt, the munition was thrown overboard by a crewmember who subsequently experienced second-degree burns and large fluid-filled vesicles on the arms, necessitating burn center hospitalization, skin grafting, and physical therapy (*3*). Delays in communication among agencies resulted in the entry of clams that had been dredged with the CWM into production. This resulted in a recall of 192 cases of clam chowder base and subsequent destruction of 704 cases of affected clams. No affected clams were distributed into commerce. Testing performed by the U.S. Coast Guard’s (USCG) Atlantic Strike Team (AST) confirmed no residual contamination aboard the vessel.

**August 2017.** A commercial fishing vessel dredged a crate of 20 sulfur mustard canisters off the coast of Long Branch. The crate broke open on the ship’s sorting belt, exposing three crewmembers. All canisters were thrown overboard using a magnet. One crewmember who disentangled a CWM from fishing gear experienced second-degree burns to the forearms and was prescribed burn cream and an oral antibiotic and advised to follow up with a burn center. The remaining two crewmembers were uninjured. After the New Jersey Department of Health was notified, approximately 5,300 bushels of purchased surf clams in 168 cages were embargoed, sanitized, destroyed, and disposed of in a landfill. Communication between the health department and the shellfish processing plant prevented transport of the clams to the plant. AST vessel testing found no residual contamination.

**October 2023.** A fishing vessel dredged a leaking CWM off the coast of Cape May, exposing two crewmembers. One crewmember threw the CWM overboard and required overnight emergency department treatment for respiratory distress and second-degree burns to the arm and neck. Treatment included supportive care, antibiotics, and guidance for treating burns. The crewmember was anticipated to make a full recovery; however, additional follow-up information is not available. The second crewmember was in the wheelhouse (the enclosed elevated control center of the boat) and experienced a burning sensation on the face but did not require medical treatment. Although no clams entered processing, delayed notification over a holiday weekend prevented prompt destruction. Approximately 32 bushels of surf clams in 22 cages were segregated, destroyed, and disposed of in a landfill. The vessel was sanitized, and AST testing found no residual contamination.

## Preliminary Conclusions and Actions

Recovered CWMs continue to pose worker and food safety risks. Because of ocean drift, storms, and offshore industries, sea-disposed CWMs locations are largely unknown and potentially far from their originally documented dump site. In the absence of knowledge about the stability of dredged CWMs, throwing these items overboard remains the safest option for fishing crews; however, this practice risks future recovery and exposure. Although U.S. law addresses hazardous materials broadly, CWMs that have remained underwater for extended periods are generally considered abandoned and degraded to the point that they are no longer treated as military weapons, and U.S. law does not require their active recovery or destruction.

Because responses to retrieved sea-disposed CWMs involve USCG, the Food and Drug Administration, state agencies, and fishing and seafood operations, responses are complex and time-consuming. Maintaining robust channels of communication among these entities might reduce risks for worker harm and prevent the occurrence of foodborne illness from sea-disposed CWMs.

Efforts to prevent CWMs encounters include avoiding documented dumping areas (*4*), engineering procedures (e.g., containment and magnet-assisted sorting), administrative measures (e.g., exposure prevention policies); and use of personal protective equipment (*5*). When a worker has a suspected CWMs encounter, safety should be prioritized by following the steps detailed in the Recognize, Retreat, and Report guidelines (*5*), including documenting the dump location. All workers should have adequate personal protective equipment and receive training in safe handling of recovered CWMs to prevent or limit exposure (Recovery of Sea-Disposed Chemical Warfare Material | CDC). Prompt care and reporting are crucial for worker health and food safety.

## Footnotes

* 45 C.F.R. part 46.102(l)(2), 21 C.F.R. part 56; 42 U.S.C. Sect. 241(d); 5 U.S.C. Sect. 552a; 44 U.S.C.
